# Does home equipment contribute to socioeconomic gradients in Australian children’s physical activity, sedentary time and screen time?

**DOI:** 10.1186/s12889-016-3419-9

**Published:** 2016-08-05

**Authors:** Dot Dumuid, Timothy S. Olds, Lucy K. Lewis, Carol Maher

**Affiliations:** 1 Alliance for Research in Exercise, Nutrition and Activity (ARENA), School of Health Sciences, University of South Australia, GPO Box 2471, Adelaide, South Australia 5001 Australia; 2 Discipline of Physiotherapy, School of Health Sciences, Faculty of Medicine, Nursing and Health Sciences, Flinders University, Adelaide, South Australia Australia

**Keywords:** Home equipment, Socioeconomic, MVPA, Sedentary, Screen time

## Abstract

**Background:**

Activity behaviours (physical activity, sedentary time and screen time) have been linked to health outcomes in childhood. Furthermore, socioeconomic disparities have been observed in both children’s activity behaviours and health outcomes. Children’s physical home environments may play a role in these relationships. This study aimed to examine the associations and interactions between children’s physical home environment, socioeconomic status and moderate-to-vigorous physical activity, sedentary time and screen time.

**Methods:**

Australian children (*n* = 528) aged 9–11 years from randomly selected schools participated in the cross-sectional International Study of Childhood Obesity, Lifestyle and the Environment. Children’s physical home environment (access to equipment), socioeconomic status (household income and parental education) and demographic variables (gender and family structure) were determined by parental questionnaire. Moderate-to-vigorous physical activity and sedentary time were measured objectively by 7-day 24-h accelerometry. Screen time was obtained from child survey. The associations between the physical home environment, socioeconomic status and moderate-to-vigorous physical activity, sedentary time and screen time were examined for 427 children, using analysis of covariance, and linear and logistic regression, with adjustment for gender and family structure.

**Results:**

The presence of TVs (*p* < 0.01) and video game consoles (*p* < 0.01) in children’s bedrooms, and child possession of handheld video games (*p* = 0.04), cell phones (*p* < 0.01) and music devices (*p* = 0.04) was significantly and positively associated with screen time. Ownership of these devices (with the exception of music devices) was inversely related to socioeconomic status (parental education). Children’s moderate-to-vigorous intensity physical activity (*p* = 0.04) and possession of active play equipment (*p* = 0.04) were both positively associated with socioeconomic status (household income), but were not related to each other (with the exception of bicycle ownership).

**Conclusions:**

Children with less electronic devices, particularly in their bedrooms, participated in less screen time, regardless of socioeconomic status. Socioeconomic disparities were identified in children’s moderate-to-vigorous physical activity, however socioeconomic status was inconsistently related to possession of active play equipment. Home active play equipment was therefore not a clear contributor to the socioeconomic gradients in Australian children’s moderate-to-vigorous physical activity.

## Background

Regular moderate-to-vigorous intensity physical activity (MVPA) mitigates children’s cardiometabolic risk and adiposity, and promotes skeletal health and psychological wellbeing [1]. Conversely, mounting evidence links high screen time to poor fitness, lower self-esteem and low academic achievement [2]. In addition, excessive screen time in childhood appears to predispose children to accelerated pathophysiology and poor health in later years [3]. Furthermore there is mixed evidence regarding the health associations of total sedentary time in childhood [4, 5].

Ecological theory identifies the home environment as a critical influence in the development of children’s health behaviours [6]. Children’s MVPA, sedentary time and screen time may be influenced by household socioeconomic status (SES), and the physical home environment, (in terms of access to equipment) and potential interactions between these factors. Recent systematic reviews on the correlates of children’s physical activity reported mixed results for the significance of SES [7, 8] and home active play equipment [9, 10]. Similarly, children’s sedentary time has not been consistently associated with SES or possession of sedentary electronic devices [11]. However, strong positive associations have been identified between screen time and both socioeconomic disadvantage and the presence of televisions (TVs) in children’s bedrooms [9–11].

The relationships between children’s physical home environment and SES, and whether such associations may underpin SES gradients in MVPA and sedentary time have, to our knowledge, only been examined in combination by a single study. Tandon et al’s study of 715 American children (aged 6–11 years) [12] suggested that low-SES households possessed less active play equipment and more electronic devices than high-SES households. In particular, children from low-income households had less play equipment items, but more electronic devices in their bedrooms, compared to children from high-income households. These findings suggest that the physical home environment in high-SES households may be more conducive to MVPA and less supportive of sedentary time than the home environment in low-SES households.

Home access to equipment has, to our knowledge, not been investigated as a potential mediator for SES disparities in children’s MVPA or sedentary time in previous research. Whilst Tandon and colleagues set out to investigate this possibility, mediation analyses for MVPA and sedentary time were not carried out because SES disparities in MVPA or sedentary time were not demonstrated in their sample [12]. However, the authors identified significant SES differences in screen time, which were strongly mediated by the presence of a TV in children’s bedrooms [12]. It remains unknown if the physical home environment mediates the socioeconomic differences in MVPA and sedentary time in populations where such SES gradients are evident.

## Methods

### Aims

This study set out to examine all three sides of this activity pattern, SES and home equipment triad, by establishing if 1) Australian children’s MVPA, sedentary time and screen time differed by SES, 2) children’s home access to active play equipment and sedentary electronic devices varied by SES and 3) the physical home environment mediated children’s MVPA, sedentary time and screen time. We hypothesized that any SES disparities in children’s activity patterns would be mediated by differences in access to home equipment. Analyses were undertaken using objectively measured MVPA and sedentary time data in a sample of 9–11 year old Australian children.

### Participants and study design

Participants for this study were from the Australian arm of the International Study of Childhood Obesity, Lifestyle and the Environment (ISCOLE), a 12-nation cross-sectional study, involving around 7000 children aged 9 to 11 years [13].

Schools in greater metropolitan Adelaide, South Australia were stratified into SES tertiles, using the schools’ ‘Index of Community Socio-Education Advantage’ (ICSEA) score [14]. Schools randomly selected from each socioeconomic tertile were invited, regardless of school size. Principals from a total of 26 schools consented to their schools’ participation in the study, with a final school participation rate of 46 %. Participating schools did not differ significantly from invited non-participating schools in terms of SES (*p* = 0.32) or total student enrolments (*p* = 0.10). School principals consented to the dissemination of study recruitment materials to all year five children enrolled at the school. Children were invited to attend an information session where information and recruitment material was sent home with children to parents. Parents returned a completed consent form in a pre-paid envelope if both they and their child agreed to be involved in the study. The child response rate was 57 %. Data collection was conducted continuously during school terms from September 2011 to December 2012, with schools from each SES tertile being involved over all seasons.

The final Australian ISCOLE sample consisted of 528 children. To be included in the ensuing analyses, complete data for home equipment (*n* = 497), as well as valid accelerometry (*n* = 464) and sociodemographic variables (*n* = 427), were required.

Ethical approval for the Australian protocol was granted by the University of South Australia Human Research Ethics Committee, the South Australian Department of Child Development, and the Catholic Education Department of South Australia. ISCOLE was registered on ClinicalTrials.gov, Identifier: NCT01722500. Written informed consent was obtained from parents or guardians, and assent was obtained from children before participation.

### Measures

Presence of equipment in the home was determined by parental responses to the ISCOLE Neighbourhood and Home Environment Questionnaire [13]. Parents reported whether their child had access at home to the following active play equipment: bike; basketball hoop; jump rope; active video games (e.g. Wii); sports equipment (e.g. ball, racquets, bats); swimming pool; rollerskates/skateboard/scooter; and fixed play equipment (e.g. swing set, play house). An ‘Active Play Equipment Score’ was generated using the sum of individual active play items (possible range 0–8). Parents also reported whether their child’s bedroom contained the following electronic items: TV; computer; or video game system (e.g. PlayStation, Xbox); and if their child had access in the home to a cell phone or 2-way radio, hand-held videogame players (e.g. Game Boy, Sony PlayStation Portable) or music systems (e.g. iPod, stereo, radio). A ‘Sedentary Electronic Equipment Score’ was generated using the sum of electronic items accessible to the child (possible range 0–6).

### Moderate-vigorous physical activity, sedentary time and screen time

MVPA and sedentary time were measured objectively by 24-h 7-day accelerometry. An Actigraph GT3X+ triaxial accelerometer on a waist belt was worn at the mid-axillary line on the right hip. To be considered valid, participants were required to have a minimum of 10 waking-hours of wear time per day for at least four days, including at least one weekend day. Previously published algorithms were used to identify the nocturnal sleep period and determine wake-wear time [15, 16]. Accelerometer data were collected at 80 Hz, and then aggregated in 15-s periods. Data were converted to average daily minutes of MVPA (≥574 counts/15 s) and sedentary time (≤25 counts/15 s), using Evenson’s age-specific cut points [17]. Mean weekday and weekend day MVPA were weighted at a ratio of 5:2. A daily MVPA index was calculated by adding the weighted means of MVPA and dividing by the sum of the weights. The same procedure was used with sedentary time to generate a daily sedentary time index.

Daily TV time and video or computer time on both an average weekday and an average weekend day was self-reported by children in the following categories: 1 = none; 2 = <1 h; 3 = 1 h; 4 = 2 h; 5 = 3 h; 6 = 4 h; and 7 = ≥5 h. For analysis, a continuous variable was created for both TV time and video/computer time as follows: none = 0 h, <1 = 0.5 h, 1 = 1 h; 2 = 2 h; 3 = 3 h; 4 = 4 h; and ≥5 = 5.5 h. A daily screen time index was then calculated by determining weekday and weekend total screen time (TV viewing plus video/computer time) and then weighting weekday and weekend screen time at a ratio of 5:2. The resultant times were added together and divided by the sum of the weights to produce a daily screen time index.

### Household socioeconomic status

Household SES was determined by parent-reported household income and highest education level. Annual gross household income was collapsed into the following Australian dollar categories: 1 = <$50,000; 2 = $50,000 to $89,999; 3 = $90,000 to $139,999; and 4 = ≥$140,000 (at the time of data collection, 1AUD was approximately 0.95USD).

The highest educational level attained by either parent was collapsed into the following categories: 1 = ≤high school; 2 = some post-high school (vocational diploma or certificate); 3 = bachelor degree; and 4 = graduate/professional degree.

### Covariates

Parents reported their child’s date of birth, gender and number of siblings and parental figures residing in the household. The number of siblings was categorised into: 1 = none; 2 = one; 3 = two; and 4 = three or more. The number of parents living at home (including biological mother, biological father, step mother, step father or legal guardian) was categorised into ≤1 parent or ≥2 parents.

### Analysis

STATA 14.0 was used for all analysis. Data were distributed normally, with the exception of MVPA and screen time. These variables were normalised by log-transformation, and square root transformation, respectively. The effect of clustering at the school level was adjusted for using STATA’s ‘svyset’ command. Exploratory analyses revealed that participants from households of disadvantaged SES (income and education) were more likely to be female, have more siblings and single parents than those of advantaged SES. Therefore gender and number of siblings and number of parents were included as covariates in all models.

The analysis consisted of testing for significant relationships between (1) levels of SES (income and education) and MVPA, sedentary time and screen time; (2) levels of SES and home access to equipment and (3) home access to equipment and MVPA, sedentary time and screen time.

Analysis of covariance was used to determine whether participants’ MVPA, sedentary time or screen time differed across SES categories. Children’s equipment was compared across different income and education groups using logistic regression for possession of individual items and linear regression for Active Play and Sedentary Electronic Equipment Scores. The association between possession of equipment and MVPA, sedentary time and screen time was determined by logistic regression for individual items and linear regression for Active Play and Sedentary Equipment Scores.

## Results

### Participant characteristics

Participants’ characteristics are shown in Table 1. Participants generally owned many items of active play equipment, however 45 % did not achieve the recommended daily level of MVPA (operationalized as < 60 min MVPA on an average day [18, 19]). They also exceeded national daily screen time recommendations by an average of 49 min (operationalized as > 2 h daily screen time on an average day [18, 19]). Fifty-eight percent reported an excess of two hours of daily screen time. Children excluded from the analysis (due to incomplete data) were more likely to be from households of lower SES (*p* < 0.01), but did not vary on the basis of any other characteristics.

**Table 1:** Sociodemographic and behavioural characteristics of participants

Characteristic		*n*; total *n* = 427 (%)
Gender, *n* (% boys)		193 (45 % boys)
Age, years (SD)		10.3 (0.5)
Parents in household, *n* (%)	≤1	74 (17)
	2	353 (83)
Number of siblings, *n* (%)	0	30 (7)
	1	195 (46)
	2	119 (28)
	≥3	83 (19)
Highest parental education, *n* (%)	≤ High school	79 (19)
	Diploma	162 (38)
	Bachelor	105 (25)
	Graduate/professional degree	81 (19)
Household Annual Income^a^, *n* (%)	< AUD50,000	86 (20)
	AUD50,000-89,999	138 (32)
	AUD90,000- 139,999	109 (26)
	AUD140,000+	94 (22)
Active play equipment score, mean (SD)		6.0 (1.5)
Sedentary electronic equipment score, mean (SD)		2.4 (1.2)
Daily MVPA index, mean min (SD)		65.3 (23)
Daily sedentary time index, mean min (SD)		476 (60)
Daily screen time index, min (SD)		169 (105)

*MVPA* Moderate to vigorous physical activity

^a^At the time of data collection, 1AUD was equal to approximately 0.95USD

### Association between SES and MVPA, sedentary time and screen time

Higher annual household income was significantly associated with higher levels of children’s MVPA (*p* = 0.04). Children in the lowest income category (<AUD50,000) performed an average of 11 min less MVPA per day than children in the highest income category (≥AUD140,000). There was no association between parental education and MVPA (*p* = 0.31). No statistically significant relationships were observed between either SES indicator and sedentary time (income: *p* = 0.68; education: *p* = 0.05), or screen time (income: *p* = 0.52; education: *p* = 0.18).

### Association between SES and home equipment

Compared to the reference category for annual household income (<AUD50,000), home access to bikes, basketball hoops and rollerskates/skateboards/scooters was higher in the highest income households (*p* = 0.03, *p* = 0.01 and *p* = 0.03 respectively) (Table 2). There was an overall trend for increased ownership of individual active play equipment items across increasing household income bands, and children from the highest income households possessed significantly more equipment than children from the lowest income households (*p* = 0.04). In general, home play equipment did not vary across parental education categories.

**Table 2:** Difference in home equipment access across income and education bands

	Household income				Highest parental education			
	<AUD50,000 Reference	AUD50,000-89,999	AUD90,000-139,999	AUD140,000+	≤ High School Reference	Trade or diploma	Bachelor degree	Graduate/ Professional degree
	*n* = 86	*n* = 138	*n* = 109	*n* = 94	*n* = 79	*n* = 162	*n* = 105	*n* = 81
Active play equipment (% yes)								
Bike	84	93*	92	96*	91	93	92	89
Basketball hoop	55	61	72	81*	57	69	69	69
Active video game	76	79	83	86	80	88	77	74
Sports equipment (e.g. ball, bat)	94	96	98	100	92	98	100	98
Swimming pool	35	45	44	51	34	50*	44	42
Skateboard, etc.	81	90*	88	90*	91	90	86	83
Skipping rope	73	75	72	66	68	74	73	68
Fixed play equipment	55	58	60	51	57	59	51	56
Total active play equipment score Mean (SD)	5.5 (1.7)	6.0 (1.5)	6.1 (1.5)	6.2 (1.2)*	5.7 (1.6)	6.2 (1.4)	5.9 (1.4)	5.8 (1.8)
Sedentary electronic equipment (% yes)								
TV in bedroom	48	38	27*	16*	53	38*	17*	19*
Computer in bedroom	12	20	19	14	18	15	16	19
Video game in bedroom	14	20	14	11	23	19	10*	7*
Own cell phone	26	25	15	16	29	23	14*	14*
Own handheld video game	77	79	78	69	86	78	73	65*
Own music device	71	80*	89*	87*	80	85	85	75*
Total sedentary electronic equipment score Mean (SD)	2.5 (1.2)	2.6 (1.3)	2.4 (1.3)	2.1 (1.2)	2.9 (1.1)	2.6 (1.3)*	2.2 (1.2)*	2.0 (1.1)*

*denotes significant difference (*p* < 0.05) from the reference category. All analyses are adjusted for child’s gender, number of siblings and number of parents

Access to sedentary electronic equipment varied across both measures of SES (Table 2). The highest prevalence of TVs in participants’ bedrooms was in both the lowest income (*p* < 0.01) and education categories (*p* < 0.01), with children from the lowest income households three times more likely to have a TV in their bedroom (48 %) than children from the highest income households (16 %). Children with parents of lower education levels also had relatively more video game consoles in their bedrooms (*p* < 0.01), and higher ownership of mobile phones (*p* = 0.04) and handheld video games (*p* = 0.02). The only electronic equipment showing the opposite trend was music devices (e.g. iPods, stereos, radios), with children from higher income households reporting higher possession (*p* = 0.01) than lower income households. There was a significant inverse association between total number of sedentary electronic items and parental education categories (*p* = 0.04).

### Association between home equipment and MVPA, sedentary time and screen time

Bicycle ownership (*p* > 0.01) was significantly associated with higher MVPA. No other individual active play equipment item was associated with MVPA, nor was the total number of active play equipment items (*p* = 0.08) (Table 3). In contrast, ownership of electronic devices [namely presence of a TV (*p* < 0.01) or video game console (*p* < 0.01) in the bedroom, ownership of a cell phone (*p* < 0.01), handheld video game (*p* = 0.04) and music device (*p* = 0.04), as well as total number of electronic items (*p* < 0.01)] was consistently associated with higher screen time. Ownership of video game consoles (*p* = 0.03), but not any other electronic devices, was associated with higher total sedentary time.

**Table 3:** Difference in MVPA, sedentary time and screen time across home access to equipment

	MVPA (mean, min/day)			Sedentary time (mean, min/day)			Screen time (mean, min/day)		
	No	Yes	*p*	No	Yes	*p*	No	Yes	*p*
Active play equipment									
Bike	54	66	<0.01*						
Basketball hoop	64	66	0.06						
Active video game	63	66	0.32						
Sports equipment (e.g. ball, bat)	55	66	0.33						
Swimming pool	64	67	0.20						
Roller skates, etc.	62	66	0.51						
Skipping rope	67	64	0.59						
Fixed play equipment	66	65	0.67						
Total active play equipment score, standardised beta (*p* value)	0.10		*p *= 0.08						
Sedentary electronic equipment									
TV in bedroom				476	476	0.80	160	187	<0.01*
Computer in bedroom				475	481	0.37	168	170	0.98
Video game in bedroom				475	484	0.03*	162	204	<0.01*
Own cell phone				477	475	0.85	161	196	<0.01*
Own handheld video game				485	474	0.10	146	176	0.04*
Own music device				479	476	0.30	155	171	0.04*
Total sedentary electronic equipment score, standardized beta				0.00		*p* = 0.09	0.02		*p* < 0.01*

Models are adjusted for child’s gender, number of parents and number of siblings. * denotes statistically significant values

### Contribution of home equipment

Access to home equipment was not a mediator of SES gradients in children’s sedentary or screen time because no SES disparities were identified in either sedentary time or screen time. In addition, home access to play equipment (with the exception of bicycles) was not linked with higher MVPA, therefore ownership of such equipment cannot be considered as a mediator for the SES gradients observed in MVPA. Bicycle ownership may contribute to SES disparities in MVPA, as possession of a bicycle was associated with both higher SES (*p* = 0.03) and higher MVPA (*p* < 0.01).

## Discussion

This study aimed to investigate the relationships and interactions between Australian children’s physical home environment, SES, and MVPA, sedentary time and screen time. A weak positive association was detected between MVPA and SES (household income only). Similarly, ownership of active play items was weakly associated with income. Despite this, ownership of active play equipment (excluding bicycles) was not associated with MVPA. Socioeconomic gradients were not detected in self-reported screen time or total sedentary time. However, strong associations were detected between electronic device ownership and SES, as well as electronic device ownership and screen time.

The finding of a weak positive relationship between children’s MVPA and SES has been observed in other developed countries [20, 21], as has the lack of association between SES and children’s overall sedentary time [22, 23]. However, the lack of SES disparities in screen time is in contrast to growing epidemiological evidence of such disparities [11, 24]. It is possible that the self-report measure of screen time used in this study introduced validity concerns because participants were at the age where the ability to recall temporal data accurately is still emerging [25].

This study identified socioeconomic inequities in children’s physical home environments, with low-income households possessing relatively less active play equipment. Surprisingly, children from low-income households owned relatively more electronic equipment than children from high-income households. Australian expenditure data support this finding, with lower SES families spending less on active recreation and more on screen recreation than higher SES families [26]. This is particularly surprising, given that electronic equipment is, generally speaking, more expensive than active play equipment. Internationally research in this field is scarce, however one American study described similar findings between SES and home equipment [12]. Furthermore, the American study concurs with the current study and previous literature that electronic devices were more likely to be located in low-SES children’s bedrooms [12, 27, 28]. The associations between lower SES and higher electronic equipment ownership were evident when SES was operationalised in terms of both parental education and income. Taken together, the findings appear to suggest that the socioeconomic differences in a child’s home equipment may be driven primarily by cultural, rather than financial factors [26].

In this study, children’s home access to electronic equipment was strongly linked with higher screen time, but not clearly linked with total sedentary time. These findings are consistent with recent research [9, 10], and add to growing evidence that screen and non-screen sedentary time are separate phenomena, each possessing unique drivers and associations [9]. As such, future research in this field should distinguish between types of sedentary behaviours. Home access to active play equipment was largely unrelated to children’s MVPA in this study, consistent with the findings of a previous systematic review [10]. It is possible that the relatively high prevalence of active play equipment in western households created a “ceiling effect”, leading to a lack of a significant relationship between equipment and MVPA.

This study had several methodological strengths. A relatively large sample of children who attended a random selection of schools throughout a large Australian city was included. Furthermore, the analysis accounted for the possible effects of clustering at the school level and potential confounders. MVPA and sedentary time were objectively measured using 7-day 24-h accelerometry, which achieved a high compliance rate.

Several limitations must be acknowledged. Firstly, recruitment of participants from a single city potentially limits the generalisability of the findings to other areas. Secondly, accelerometers are known to under-report the intensity of activities such as cycling, skateboarding, riding scooters and rollerblading. As the ownership of these items was significantly higher for children from higher SES households, it is possible that the difference in MVPA across SES is more significant than our results indicate. In addition, sedentary time measured by waist-worn accelerometers does not accurately distinguish between sitting and standing time [29]. Thirdly, the cross-sectional nature of the study means causation cannot be inferred. Finally, there have been rapid advances in the use of technology since the questionnaires in this study were developed. A recent Australian study, for example, found that 58 % of children surveyed used iPads or tablets on weekdays [30]. The home equipment questionnaire used in this study was not sensitive to these newer devices.

There is mounting evidence that Australian children do not meet the recommended national guidelines for screen time. In the present study, 58 % of children reported over two hours of daily screen time on an average day. The same rate of non-adherence (58 %) on an average day was reported in a previous study of Australian adolescents aged 11–13 y [19].

Given the adverse outcomes associated with screen time, there is some urgency to identify potentially modifiable correlates of children’s screen time. This study found that home access to electronic equipment, especially in children’s bedrooms, was a significant correlate of higher screen time. However, as this finding does not imply causation, caution must be implemented when advocating the removal of electronic devices from children’s bedrooms as a strategy to reduce screen time.

A large proportion of Australian children fail to meet daily physical activity guidelines, with one previous study finding that 43 % of 11–13 y olds did not achieve ≥ 1 h MVPA on an average day [19]. In the present study, a comparable proportion of children (45 %) did not meet the Australian physical activity recommendations on an average day*. *Children from low-SES households performed less MVPA and possessed less active play equipment than children from high-SES households. Therefore, it could be hypothesised that increasing home access to play equipment may mitigate the SES disparities in MVPA. However, this study found that, with the exception of bicycles, access to home play equipment was unrelated to MVPA. Bicycle ownership may contribute to the SES differences in MVPA, and should be further investigated in intervention studies. Future research should investigate the contribution of other potential mediators of SES disparities, for example, the social environment within children’s homes (household chaos, parenting styles) or neighbourhood factors (perceived safety, availability of recreational facilities). Furthermore, future studies could examine the relationships between SES, children’s home environment and MVPA in different countries around the world.

## Conclusions

Children’s ownership of electronic equipment was positively associated with screen time and inversely related to SES. However, children’s sedentary time and screen time did not differ across SES. The socioeconomic disparities in children’s MVPA were inconsistently related to home access to active play equipment.

## Abbreviations

AUD, Australian dollar; ICSEA, Index of Community Socio-Education Advantage; ISCOLE, International Study of Childhood Obesity, Lifestyle and the Environment; MVPA, moderate-to-vigorous physical activity; SES, socioeconomic status; TV, television; USD, United States dollar

## References

[1] Janssen I, LeBlanc AG (2010). Review Systematic review of the health benefits of physical activity and fitness in school-aged children and youth. Int J Behav Nutr Phys Act.

[2] Tremblay MS, LeBlanc AG, Kho ME, Saunders TJ, Larouche R, Colley RC, Goldfield G, Gorber SC (2011). Systematic review of sedentary behaviour and health indicators in school-aged children and youth. Int J Behav Nutr Phys Act.

[3] Sigman A (2012). Time for a view on screen time. Arch Dis Child.

[4] Tremblay MS, Colley RC, Saunders TJ, Healy GN, Owen N (2010). Physiological and health implications of a sedentary lifestyle. Appl Physiol Nutr Metab.

[5] Chinapaw M, Proper K, Brug J, Van Mechelen W, Singh A (2011). Relationship between young peoples’ sedentary behaviour and biomedical health indicators: a systematic review of prospective studies. Obes Rev.

[6] Davison KK, Birch LL (2001). Childhood overweight: a contextual model and recommendations for future research. Obes Rev.

[7] Uijtdewilligen L, Nauta J, Singh AS, van Mechelen W, Twisk JW, van der Horst K, Chinapaw MJ (2011). Determinants of physical activity and sedentary behaviour in young people: a review and quality synthesis of prospective studies. Br J Sports Med.

[8] Ferreira I, Van Der Horst K, Wendel‐Vos W, Kremers S, Van Lenthe FJ, Brug J (2007). Environmental correlates of physical activity in youth–a review and update. Obes Rev.

[9] Kaushal N, Rhodes RE (2014). The home physical environment and its relationship with physical activity and sedentary behavior: A systematic review. Prev Med.

[10] Maitland C, Stratton G, Foster S, Braham R, Rosenberg M (2013). A place for play? The influence of the home physical environment on children’s physical activity and sedentary behaviour. Int J Behav Nutr Phys Act.

[11] Temmel CS, Rhodes R (2013). Correlates of sedentary behaviour in children and adolescents aged 7-18: A systematic review. Health Fitness J Canada.

[12] Tandon PS, Zhou C, Sallis JF, Cain KL, Frank LD, Saelens BE (2012). Home environment relationships with children’s physical activity, sedentary time, and screen time by socioeconomic status. Int J Behav Nutr Phys Act.

[13] Katzmarzyk PT, Barreira TV, Broyles ST, Champagne CM, Chaput J-P, Fogelholm M, Hu G, Johnson WD, Kuriyan R, Kurpad A (2013). The international study of childhood obesity, lifestyle and the environment (ISCOLE): Design and methods. BMC Public Health.

[14] My School: Guide to understanding 2012 index of community socio-educational advantage (ICSEA) values [http://www.acara.edu.au/verve/_resources/About_icsea_2014.pdf]. Accessed 15 Mar 2016.

[15] Tudor-Locke C, Barreira TV, Schuna JM, Mire EF, Chaput J-P, Fogelholm M, Hu G, Kuriyan R, Kurpad A, Lambert EV (2015). Improving wear time compliance with a 24-h waist-worn accelerometer protocol in the International Study of Childhood Obesity, Lifestyle and the Environment (ISCOLE). Int J Behav Nutr Phys Act.

[16] Tudor-Locke C, Barreira TV, Schuna JM, Mire EF, Katzmarzyk PT (2013). Fully automated waist-worn accelerometer algorithm for detecting children’s sleep-period time separate from 24-h physical activity or sedentary behaviors. Appl Physiol Nutr Metab.

[17] Evenson KR, Catellier DJ, Gill K, Ondrak KS, McMurray RG (2008). Calibration of two objective measures of physical activity for children. J Sports Sci.

[18] Australia's physical activity and sedentary behaviour guidelines [http://www.health.gov.au/internet/main/publishing.nsf/Content/health-pubhlth-strateg-phys-act-guidelines]. Accessed 15 Mar 2016.

[19] Olds T, Ridley K, Wake M, Hesketh K, Waters E, Patton G, Williams J (2007). How should activity guidelines for young people be operationalised?. Int J Behav Nutr Phys Act.

[20] Maher CA, Olds TS (2011). Minutes, MET minutes, and METs: unpacking socio-economic gradients in physical activity in adolescents. J Epidemiol Community Health.

[21] Drenowatz C, Eisenmann JC, Pfeiffer KA, Welk G, Heelan K, Gentile D, Walsh D (2010). Influence of socio-economic status on habitual physical activity and sedentary behavior in 8-to 11-year old children. BMC Public Health.

[22] King AC, Parkinson KN, Adamson AJ, Murray L, Besson H, Reilly JJ, Basterfield L, Team GMSC (2011). Correlates of objectively measured physical activity and sedentary behaviour in English children. Eur J Pub Health.

[23] Kelly LA, Reilly JJ, Fisher A, Montgomery C, Williamson A, McColl JH, Paton JY, Grant S (2006). Effect of socioeconomic status on objectively measured physical activity. Arch Dis Child.

[24] Cillero IH, Jago R (2010). Systematic review of correlates of screen-viewing among young children. Prev Med.

[25] Friedman WJ (2007). The development of temporal metamemory. Child Dev.

[26] Aitken R, King L, Bauman A (2008). A comparison of Australian families’ expenditure on active and screen‐based recreation using the ABS Household Expenditure Survey 2003/04. Aust N Z J Public Health.

[27] Hardy LL, King L (2012). Weight and weight related behaviours among NSW Kindergarten children. University of Sydney.

[28] Gilbert-Diamond D, Li Z, Adachi-Mejia AM, McClure AC, Sargent JD (2014). Association of a television in the bedroom with increased adiposity gain in a nationally representative sample of children and adolescents. JAMA Pediatr.

[29] Pedišić Ž (2014). Measurement issues and poor adjustments for physical activity and sleep undermine sedentary behaviour research—the focus should shift to the balance between sleep, sedentary behaviour, standing and activity. Kinesiology.

[30] Houghton S, Hunter SC, Rosenberg M, Wood L, Zadow C, Martin K, Shilton T (2015). Virtually impossible: limiting Australian children and adolescents daily screen based media use. BMC Public Health.

